# Exploring the views of being a proxy from the perspective of unpaid carers and paid carers: developing a proxy version of the Adult Social Care Outcomes Toolkit (ASCOT)

**DOI:** 10.1186/s12913-019-4025-1

**Published:** 2019-03-29

**Authors:** James Caiels, Stacey Rand, Tanya Crowther, Grace Collins, Julien Forder

**Affiliations:** 1 Quality and Outcomes of person-centred care policy Research Unit (QORU), PSSRU Kent, University of Kent, Cornwallis Building, Canterbury, CT2 7NF UK; 2 NatCen Social Research, 35 Northampton Square, London, EC1V 0AX UK

**Keywords:** Quality of life, Social care, Outcomes, ASCOT, Proxy

## Abstract

**Background:**

Outcomes-based policy and administration of public services present a compelling argument for the value of outcomes data. However, there are a number of challenges inherent in collecting these data from people who are unable to complete a paper-based survey or interview due to cognitive or communication impairments. In this paper, we explore the views of being a proxy from the perspective of unpaid carers and paid carers who may be asked to act as a proxy on behalf of the person(s) they care for. We consider the key issues that need to be addressed when adapting an instrument designed to measure social care outcomes, the Adult Social Care Outcomes Tool (ASCOT), into a proxy-report tool.

**Methods:**

Participants took part in either a focus group (35 paid carers in eight focus groups), or a one-to-one interview (eight unpaid carers). All participants were recruited via carer organisations and care providers. Transcripts, field notes and audio data collected during focus groups and interviews were analysed using a thematic framework approach.

**Results:**

Participants agreed that any person acting as a proxy would need to be very familiar with the care recipient, as well as their needs and care provision. A number of provisions for proxy respondents were proposed to improve face validity and acceptability of completing a questionnaire by proxy, and to ensure that any potential bias is reduced in the design of the questionnaire. These included: providing two sets of response options for each proxy perspective (the proxy themselves and the proxy view of how they think the care recipient would respond); a comments box to help people explain why they have selected a given response option (especially where these indicate unmet need); and providing clear guidance for the proxy respondent on how they should complete the questionnaire.

**Conclusions:**

This study has shown some of the challenges involved in assessing outcomes by proxy and explored some potential ways these can be mitigated. The findings highlight the benefits of developing and testing proxy measures in a robust way to widen participation in social care research.

**Electronic supplementary material:**

The online version of this article (10.1186/s12913-019-4025-1) contains supplementary material, which is available to authorized users.

## Background

The use of self-reported outcomes in evaluating public services has become more prevalent in recent times [1], while utilising outcomes data has become increasingly important in policy-based decision making in the UK [2]. In part, its purpose is to make public services more accountable to the people that use them. The mechanism applied, points stakeholders involved in care provision toward concentrating on the principle aim of providing services – to improve people’s outcomes. In addition, identifying common factors in ‘good’ provision of public services can potentially be shared and applied to other provider settings. This outcomes-based approach has influenced different public services, including publicly-funded social care. (Social care refers to a range of different long-term care services for people with physical or intellectual disabilities, mental health problems, frailty, or impairments due to older age: for example, day care, home care or residential care).

Since social care interventions aim to support people to maintain or improve their quality of life, the quality of life of the person who receives support has been identified as a key outcome of the quality and effectiveness of social care [3–7]. In the UK, social care policy strategy has identified the quality of life of people with support needs as an overarching indicator of the performance of the social care sector [8]. A measure of social care-related quality of life, and the focus of this paper, is the Adult Social Care Outcomes Toolkit (ASCOT) [9, 10]. The ASCOT is a measure designed for self-completion by social care service users, regardless of the reason(s) why they need social care support. It is included as an overarching outcome measure in the Adult Social Care Outcomes Framework (ASCOF) for England, which seeks to provide an overview of national and local performance that promotes transparency, accountability and service improvement driven by the needs of people who use services [11]. The ASCOT questionnaire includes eight items for each of the following social care-related quality of life attributes: *Control over daily life; Occupation (‘doing things I value and enjoy’); Social participation and involvement; Personal safety; Personal cleanliness and comfort; Food and drink; Accommodation cleanliness and comfort;* and *Dignity* (www.pssru.ac.uk/ascot) [10]. Each attribute is rated by self-report as the ideal state, no needs, some needs or high-level needs [10]. In England, the ASCOT has been used to evaluate and inform policy strategy, commissioning and care practice [11–15]. Table 1 shows the ASCOT domains and definitions.

**Table 1:** ASCOT domains and definitions

Control over daily life	The respondent is able to choose what to do and when to do it, having control over daily life and activities.
Personal cleanliness and comfort	The respondent feels personally clean and comfortable and looks presentable. At best, is dressed and groomed in a way that reflects personal preferences.
Food and drink	The respondent feels that s/he has a nutritious, varied and culturally appropriate diet with enough food and drink, at regular and timely intervals, that he/she enjoys.
Personal safety	The respondent feels safe and secure. This means being free from fear of abuse, falling or other physical harm and fear of being attacked or robbed.
Social participation and involvement	The respondent feels content with his/her social situation, where social situation is taken to mean the sustenance of meaningful relationships with friends and family, and feeling involved or part of a community, should this be important to the service user.
Occupation	The respondent is sufficiently occupied in a range of meaningful activities whether it be formal employment, unpaid work, caring for others or leisure activities.
Accomodation cleanliness and comfort	The respondent feels that the home environment, including all rooms, is clean and comfortable.
Dignity	The psychological impact of the way support and care services are provided on the service user’s personal sense of significance and sense-of-self.

Copyright © 2018 University of Kent: Reproduced with permission. All rights reserved

While the rhetoric of outcomes-based policy and administration of public services presents a compelling argument for the value of outcomes data, there are challenges associated with its measurement, collection and application [1, 2]. The use of patient-reported outcome measures in the evaluation of healthcare interventions also share many of these challenges. In this paper, we consider the measurement challenge of how to collect information from people who are unable to complete a paper-based survey or interview, even with adaptations or adapted questionnaire formats, due to cognitive or communication impairments. Specifically, we focus on people with intellectual disability and/or autism, dementia or other age-related cognitive or communication impairments. The systematic exclusion of people who are unable to self-report their quality of life may contribute to issues of sample size, missing data, bias, equity and inclusion in the context of the evaluation of health and social care interventions [16, 17]. In the application of quality of life data to an outcomes-based approach that seeks to give people an active voice in shaping the public services they use, the issues of equity and inclusion are especially important.

The use of proxy respondents is one potential solution to this problem and is commonly used in health research. A proxy is an individual who reports on behalf of a study participant. Proxy report, however, also presents a number of measurement-related challenges. Although studies that compare proxy-report to self-report may not extrapolate to cases where the individual is unable to self-report, there is evidence that proxy respondents systematically underestimate quality of life compared to self-report (for example, in studies of people with dementia [18–36], stroke [37–39] or intellectual disabilities [40, 41]). This difference between proxy- and self-report has been found to be related to various factors: for example, the measurement properties of the instrument, whether the attributes are ‘objective’ or ‘subjective’, the study’s sample size, the proxy’s level of literacy, whether the proxy experiences depression or pain, and the nature and closeness of the relationship between the proxy and the individual [21, 42]. There is also evidence that proxy-rating of quality of life may vary by whether the proxy answers are based on their own view (proxy-proxy perspective) or the proxy’s internal reconstruction of what that individual may think (proxy-patient perspective), which may introduce bias if it is not considered in the presentation and wording of items [43–45].

A further consideration is whether proxy-report may inadvertently contribute to the issue of the exclusion of individuals with cognitive and communication impairments from health and social care research it is designed to address. The inappropriate use of proxy report when data collection by self-report is feasible (for example, using alternative formats such as easy-read) would contribute to the exclusion of people from ‘having a voice’. Despite these challenges with proxy-report, however, it may be argued that proxy-report is preferred to systematic exclusion of people who are unable to self-report quality of life [16, 17].

Although proxy-report is widely-used, it is notable that many quality of life instruments completed by proxy were not specifically developed as proxy-report instruments. Also, little is known about the acceptability of care-related quality of life data collection to paid or unpaid paid carers acting as proxy respondents on behalf of adults with social care support needs. While it may be argued that adaptation of standard self-completion questionnaires may enhance their acceptability and face validity for use with proxies (for example, incorporating comments boxes for proxy respondents to complete), proxy-report instruments are typically adapted and developed from self-report measures without qualitative evidence from potential proxy respondents [46].

This study is a qualitative study to identify and explore the key issues associated with acting as a proxy to report care-related quality of life from the perspective of potential proxy respondents. We wanted to explore the perceived challenges of acting as a proxy respondent, and what kinds of adaptations might mitigate these difficulties. Although others may act as proxy respondents – for example, advocates, volunteers or health care professionals – we explored the views of being a proxy from the perspective of unpaid carers and paid carers who may be asked to act as a proxy on behalf of the person(s) they care for. We aimed to assess the significance of the issues outlined above when using proxies and in turn the implications for adapting an instrument designed to measure social care outcomes, specifically the ASCOT, into a proxy-report tool. In doing this, we wanted to explore the feasibility of developing a proxy version for people with intellectual, cognitive and/or communication impairments and identify what form any adaptations may take.

## Methods

### Design

We took an inductive approach led by participants, seeking to elicit views about the challenges associated with acting as a proxy. Participants took part in either a focus group (paid carers) or a one-to-one interview (unpaid carers). This approach was primarily pragmatic to gain access to participants. Initial attempts to arrange focus groups or one-to-one interviews with all participants proved challenging due to individual circumstances and commitments. Paid carers were more able to participate in a focus group at a central office after shifts, and arranging one-to-one interviews with unpaid carers in their own homes was far more convenient to accommodate their own schedule. Both groups were presented information in the same way to maintain consistency.

**Fig. 1:**
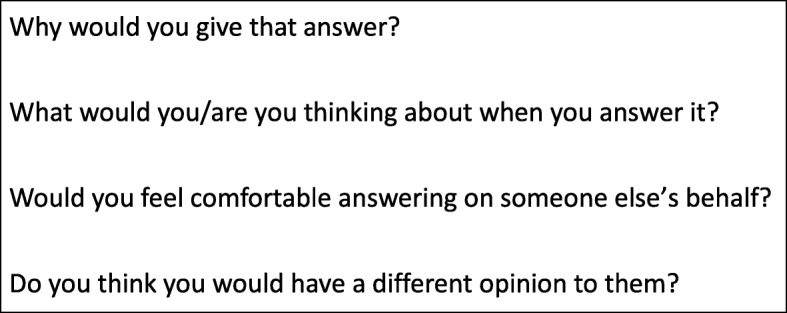
Excerpt from topic guide for focus groups and interviews

Participants in focus groups and interviews were shown the ASCOT self-completion (SCT4) questionnaire. This was not adapted in any way for proxy-use beforehand. Using a topic guide designed for this study (Additional file 1), participants were asked to comment on the questions, to think about them in relation to a (or the) person they care for, as well as consider how being a proxy might affect their response to the questions, and what adaptations would be necessary for proxy use (see Fig. 1). The ASCOT domains were presented to participants one at a time.

### Recruitment

#### Focus groups with paid carers

Paid carers were recruited to the study by contacting a randomly-selected sample of domiciliary care providers in Kent and Medway. Following initial contact with 121 care providers, individual recruitment packs with information sheets and consent forms were sent to four care providers that responded and were willing to take part. In total, 35 paid carers volunteered to participate. Eight focus groups were conducted on premises belonging to the organisations, at their request, with between two and eight participants in each.

Focus groups lasted between 75 and 90 min and each covered three or four of the eight domains of the ASCOT SCRQoL indicator. Each domain was tested a minimum of three times across the eight groups. It was not possible to present all eight domains in each group due to time constraints. Seven focus groups were recorded and transcribed verbatim, with participants’ consent. One focus group was not recorded at the request of participants; in this case, detailed notes were made.

#### Interviews with unpaid carers

A total of eight unpaid carers were recruited via carer organisations and care providers. In addition, advertisements were placed on a University Adult Research Unit mailing list and on the University staff intranet. Interviews took place in a location convenient for each interviewee, either the respondent’s home (*n* = 6) or university premises (*n* = 2).

Participants provided varying levels and types of care, although all could comment on the ASCOT attributes. Interviews lasted between 45 and 60 min and covered between three and eight SCRQoL attributes. All eight interviews were recorded and transcribed verbatim.

#### Analysis

Transcripts, field notes and audio data collected during focus groups and interviews were transferred to NVivo 10 for analysis using a framework approach [47, 48]. Framework analysis is a method for thematic analysis of qualitative data that follows a systematic series of distinct steps: transcription; familiarisation with the data; initial coding; developing an analytical framework by identifying recurrent and important themes; applying the analytical framework by indexing existing codes; charting data into a framework matrix; and interpreting the data [49]. In this study, the audio recordings from interviews and focus groups were transcribed verbatim. Two researchers (TC, JC) familiarised themselves with the transcripts, audio recordings and interview notes in order to identify key ideas and recurrent themes. Some themes were pre-defined, having been identified in the literature as potentially key issues for proxy respondents: for example, the different perspectives that proxies may adopt to answer proxy-report questions, and the acceptability of being a proxy [46, 50]. Other themes emerged through the data. The thematic framework, or index, was agreed by the researchers (JC, TC) and applied to the data. The process of coding and interpretation was influenced by the original research objective, as well as by the themes that emerged from the data. Two focus groups and two interviews were coded by both researchers. Any differences were discussed until a consensus was reached. Any subsequent cases where coding was uncertain were also discussed until consensus was reached. In the final step, we examined the range and nature of experiences and looked for associations between themes to explore explanations and practical implications.

#### Ethics

The study was reviewed and approved by the national Social Care Research Ethics Committee (SCREC) in England (now as per the NHS Health Research Authority (HRA) process) (reference: 13/IEC08/0020). Written informed consent was obtained from all participants prior to interview or focus group.

## Results

A total of 43 participants took part, with a mean age of 42 years (range 19 to 78 years). Of these, 35 were female and eight were male. Forty-one participants described their ethnicity as white British. All participants reported caring for someone with an intellectual disability and/or autism and/or dementia and/or communication or cognitive impairments related to older age (see Table 2 below).

**Table 2:** Characteristics of care-recipients known to the proxy respondents

	Overall Sample	
	N (=43)	%
Intellectual disability and/or autism	24	56
Intellectual disability and/or autism and/or dementia or Alzheimer’s	10	23
Dementia or Alzheimer’s	5	12
Age-related impairments	4	9

A number of themes emerged from the process of conducting focus groups and interviews with paid carers and unpaid carers. Those covered in this paper include: being a proxy; who can/should be a proxy; thinking and feeling on someone’s behalf; elaborating and justifying answers; differing responses; perceived impact of need and receiving care; and answering in context.

### Being a proxy

The concept of acting as a proxy was initially challenging for paid carers. Almost all paid carers reported having difficulty with the idea of answering questions on someone’s behalf, particularly when related to receiving social care services. Many reflected on their own training and how this encouraged and stipulated that all aspects of care given should actively include the care recipient as far as this was possible. Acting as a proxy appeared to be counter to that: “*I mean I wouldn’t personally complete this for anybody…not if they couldn’t provide me with any input at all I couldn’t, and I wouldn’t advise any other staff to either*” (paid carer).

By contrast, all unpaid carers were comfortable with adopting the proxy role. Unpaid carers largely saw this as an extension of their caring responsibilities where people were not able to answer for themselves: “*it’s got to be people in my position who answer on their behalf”* (unpaid carer). This level of comfort and ease with the idea of being a proxy, and answering on behalf of somebody else, appeared to stem from the nature of the relationship between the proxy and cared-for person (i.e. professional/client or family relationship). Because of this, unpaid carers could draw on their knowledge of the cared-for person and, in the case of people receiving care due to progressive disease, reflect on what had been their personal preferences prior to receiving care. This appeared to create added comfort in the proxy role for unpaid carers, and confidence that responses would be an accurate reflection of the cared for person’s view.

“*Yeah. Again, because I see them regularly and I speak to them daily, in my situation I feel that I know them quite well so I could quite easily go, yeah, that’s where she would tick sort of thing.*” (unpaid carer).

### Who can/should to be a proxy?

A critical question about using proxy respondents is who should or can act as a proxy for someone else. All respondents (paid and unpaid carers) agreed that whoever is acting as a proxy by answering questions on behalf of someone else should know that person well and be familiar with their care needs and the services that they use: “*You’d have to make sure that the person that knew that individual the most and worked with them would complete the document, wouldn’t you”* (paid carer). Even where this was the case, some carers felt they might not be able to comment on all aspects of a person’s quality of life. This would depend on a number of factors, such as the client’s specific needs or experience of living with a long-term condition, the nature and quality of the carer’s relationship with the cared-for person and the proximity of the interview to events like falls or issues with quality of care.

### Thinking and feeling on someone’s behalf

One major difficulty reported by all (paid and unpaid) carers was being required to answer questions about how the care recipient would think or feel about certain aspects of their lives. This was particularly the case for those domains that relate to abstract concepts (i.e. dignity, occupation, control over daily life).

“*I don’t think anyone would feel comfortable either trying to guess how someone feels, because I think you’d feel a bit like that’s my opinion, do you know what I mean, like, and I don’t know if I would feel right saying that’s how they feel sort of thing ‘cause I don’t really know, I can guess because obviously you do have a good relationship with them and you probably do get the gist of what they would feel, but I don’t know if I’d want to put my name to that sort of thing, do you know what I mean?*” (paid carer).

Interestingly, regardless of domain, the ASCOT questions relate to subjective experience rated by the extent to which individual needs and preferences are met. Nonetheless, questions based around more ‘tangible’ aspects of lives, such as food and drink, were generally considered less problematic.

### Elaborating and justifying answers

When considering what would help or encourage carers to answer questions as a proxy, almost all paid carers were in favour of the idea of providing an additional (optional) comments box. The purpose of this would be for proxy respondents to give additional information to the response they had chosen that they felt was relevant. Paid carers expressed that this would be particularly important in cases where the response they had chosen was towards the lower end of the scale (worst outcome states). In these cases, paid carers viewed a comments box as being essential in order to feel comfortable in answering. Paid carers envisaged using the comments box to provide further information to explain and justify their response. There was particular concern among paid carers that a bottom-level response would reflect badly on themselves, when in fact there was a ‘legitimate’ reason that they answered at this level. This could be, for example, a condition-specific behaviour that led to being unclean rather than being an indication of neglect or lack of care provision.

*“I think a massive thing that would be brilliant for, if it’s for people like us, always have comment boxes, always, like, ‘cause like you were just saying, like if you were to tick like I don’t feel at all clean and presentable, at least you’d feel a bit like, I can write why”* (paid carer).

Paid carers expressed the fear that answering negatively may result in being reprimanded or even losing their jobs. As a result, while none of the participants stated that they would deliberately answer dishonestly, this was identified as a potential issue. Almost all paid carers agreed that the provision of a comments box would allay this fear and encourage paid carers to be open and honest when answering. By contrast, unpaid carers suggested that some people may intentionally choose high needs (less positive answers) in the hope of improving services for the person they cared for. All unpaid carers though, were also in favour of a comments box in order to elaborate answers and provide more detail to justify responses.

### Differing responses

Participants reported that a major challenge of completing a questionnaire as a proxy respondent is the potential difference between how they (the carer) would respond compared to how they think the cared-for person would respond. Reasons for this include differences in personal preference, the impact of the long-term condition on an individual’s behaviour, cognition, and sense of self. An example of this was seen in the accommodation comfort and cleanliness domain, where one person’s idea of ‘clean and comfortable’ may differ from another’s because of differing standards and preferences.

*“All I would say with some of these questions, is that they could be seen different by different people, because everyone’s got different standards of cleanliness. You’ve got people that over-tidy and then might say actually my house is not tidy, but it is, or it might be the other way”* (paid carer).

Many participants, and in particular paid carers, felt that responses to the items on accommodation or personal cleanliness and food/drink would be influenced by their own views and attitudes towards how clean or tidy they keep their own house, their own preferred frequency of bathing and standards of personal hygiene, the types of food they like to eat, and so on. There was a feeling of unease in responding to questions where participants thought that their own and the cared-for person’s view, attitudes and preferences would be different. A potential solution proposed by the study participants was to include two sets of response options: one for ‘how I think this person would answer’ and another for ‘my opinion’. This was supported particularly by paid carers as a way of alleviating any concerns they had, and provided a way of expressing both perspectives.

Striking a balance between encouraging someone to make their own choices and have control over their life, against being responsible for that person’s health and wellbeing, was also something that paid carers in particular found challenging when choosing a response. For example, one paid carer explained that the person they cared for would have an extremely unhealthy and inappropriate diet if they did not help them make some healthy food choices. While it may not be what the person wanted, the carer felt it was their responsibility to manage diet and to avoid unhealthy foods. However, in considering how the person they were answering for may feel about this, paid carers considered whether this may feel like a lack of control for the person being cared for, because they were stopping someone from doing something they would otherwise choose to do. Paid carers explained that this was well-intentioned on their part and part of their responsibility as carers, but may still result in these feelings on the part of the person being cared for. Approximately half of unpaid carers also pointed to this (striking a balance) as a potential difficulty when answering the ‘control over daily life’ domain question. Almost all carers (paid and unpaid) stated that having two sets of response options would allow this to be reflected in responses.

*“He would go and sit in Tesco and eat every caramel bar and drink every bottle of Dr Pepper in there until it was all cleared out, but that isn’t going to be very good for his health so I can’t let him do that. But then he’d probably say ‘I’m not getting all the food and drink that I want’, because he’s got this obsession”* (paid carer).

### Perceived impact of need and receiving care

For some carers the very nature of being a care recipient negated being able to choose the top-level responses. Primarily this was for the ‘higher-level’ domains of control over daily life, dignity, and occupation, although for paid carers it was exclusive to the control over daily life domain. A number of paid carers argued that an individual cannot possibly have ‘as much control as they want’ (the ‘ideal state’ for the ASCOT control domain) if they are in receipt of social care services. This was predicated on the assumption that the need for any help immediately discounted the prospect of responding at the top level. ASCOT works on the principle that this is feasible due to the adaptation of an individual’s perspective, attitude and preferences to their personal circumstances. This view was not shared by all paid carers, while two unpaid carers also held this view.

*“I think as soon as you have care then it’s got to be I have adequate control over my life, because as soon as you’re having somebody come into your home, you know, you’re having to ask somebody else to do something for you at a set time, so they can’t--, they haven’t got complete control over their life”* (paid carer).

For two unpaid carers this also applied to the dignity domain, where people felt that the need for care, particularly personal care, immediately discounted the top-level response option. Again, this was based on the idea that a person ‘cannot have dignity’ if they require help with personal care: *“How can you be dignified if somebody else is wiping your bum? Sorry, but, you know, there’s nothing dignified in some of the things, is there, so it’s really not”* (unpaid carer).

### Answering in context

For some unpaid carers, timing was an important factor in how they would potentially answer. Answers may differ depending on context and what had been happening recently. Unpaid carers that identified this as a potential difficulty suggested that some guidance around the time-frame to think about when considering responses would help. When asked what period this should cover, 3 months was considered a reasonable time to reflect on.

A number of unpaid carers explained that thinking about other variables also made it difficult to aggregate in order to choose a single response. These included: which service should people be thinking about; the different aspects of domains, such as being inside or outside the home for safety; and varying levels of occupation depending on different days or different activities.

There was some concern around underestimating the impact of services, or indeed their own caring role. Participants wanted it to be clear that current quality of life was only being achieved because of the role that either paid or unpaid care played in the person’s wellbeing.

*“I would say ‘I get all the food and drink I like when I want’, but I think that gives a false impression, you know, that’s what I felt before [with previous questions], that it’s always going to give a false impression because he only gets it because there’s somebody there to give it to him, you know, otherwise he wouldn’t get anything”* (unpaid carer).

## Discussion

This qualitative study sought to identify and explore key challenges with rating ASCOT social care-related quality of life on behalf of someone else from the perspective of two groups of proxy respondents: paid carers and unpaid carers. We aimed to assess how, given the issues identified, quality-of-life instruments might be adapted for proxy use. We argue that ‘framing questions’ in a proxy instrument may help to reduce bias that can arise from respondents systematically adopting different proxy perspectives in formulating their response, and improve the acceptability of the instrument to proxy respondents. In particular, we propose that proxy questionnaires should have the following features: (1) providing two sets of response options for each proxy perspective (one for the proxy’s own view and one from the proxy’s view of what they think the care recipient would answer if they were able to); (2) adding comments boxes to help people explain why they have selected a given response option (especially where these indicate unmet need); (3) clearly identifying the role of the proxy respondent (paid or unpaid carer), including their relationship with the care recipient; and (4) providing clear guidance setting out exactly what is expected of the proxy respondent and how they should complete the questionnaire. As to the latter, this might include specific commitments that individuals’ responses would be anonymised and not linked specifically to service eligibility/assessment for individuals. We outline how we subsequently embedded these features into a proxy version of the ASCOT measure in a separate paper [51].

Some respondents, especially paid carers, expressed reservations about answering social care-related quality of life questions on someone else’s behalf. The ASCOT items all relate to subjective quality of life attributes rated against the individual’s preferred ‘ideal state’. In particular, it was noted that questions that relate to abstract areas of life that may not be directly observed were described as most difficult for the respondents to judge and respond to. Attributes like food and drink, for example, may be rated based on the respondent’s observations of the care recipient. In that case, the care recipient’s internal subjective state could be extrapolated from observable cues taken from external behaviours and expressions. By contrast, the respondents found it more difficult to rate attributes like dignity, which is an internal mental construct (i.e. the psychological impact of how care is provided), for which there may be fewer or no observable external cues for the proxy respondent to draw upon. This finding is consistent with studies that have found a higher degree of agreement between proxy-report and self-report for observable quality of life attributes (e.g. physical mobility, self-care, usual activities) compared to non-observable or subjective attributes (e.g. pain, anxiety, depression, family relationships) [17, 25, 40, 52–58].

Both paid and unpaid carers identified and explored the issue of biased ratings of quality of life by proxy respondents. Paid carers who took part in focus groups suggested that, while they themselves would not do it, other ‘unscrupulous’ carers might intentionally give more positive responses to avoid ones that may reflect badly on their care practice. This was also identified as a potential issue by unpaid carers, albeit in reverse (that is, intentionally rating lower or more negative quality of life to prompt quality improvement or increased levels of support for the care recipient). Interestingly, while there is evidence from other studies that healthcare professionals rate proxy-reported quality of life higher than unpaid carers [16, 59–61], this difference may be due to the level of contact with and closeness to the care recipient rather than a reporting bias attributable to a difference between paid and unpaid carers per se [30, 62]. The motivation to deliberately skew the overall quality of life score may be specific to data collections, such as the ASCS, which will be used to evaluate and inform policy strategy, commissioning, resource allocation or the practice of care.

Another theme identified by respondents was that proxy respondents felt that their own response would sometimes differ to how they thought the cared-for person would answer. Generally, this was either because: the proxy had differing standards or preferences (e.g. for cleanliness) from the person they were answering for; they felt happier expressing their own opinion rather than someone else’s; or that, while the cared-for person may be happy with their care provision, the proxy respondent felt improvements could be made. This difficulty may stem from uncertainty around which perspective proxy respondents should adopt in the absence of instructions (that is, should they report what they think the individual would respond (‘proxy-patient’ perspective) or their own view of the individual’s quality of life (‘proxy-proxy’ perspective) [45]). Giving detailed guidance in the instructions to the proxy respondent may go some way to addressing this issue.

The findings presented here suggest that the ‘acceptability’ of being a proxy (to the proxy) is dependent on their relationship with the cared for person and having knowledge of their needs and preferences. One implication of the findings is that while both proxy views should be regarded as valid (proxy-proxy and proxy-patient), it should be made clear which view you are asking for (collecting) and then subsequently analysing and presenting. Another implication is that we might expect less reliable answers from proxies relating to the ‘higher-order’ domains of ASCOT, and this should be acknowledged in the analysis of proxy data. Moreover this suggests that more ‘framing’ adaption is required for questions relating to attributes of quality of life perceived to be ‘less tangible’.

Interestingly, in social care data collections like the Adult Social Care Survey (ASCS) in England, only 5.9% of proxy respondents were paid carers [63]. By comparison, over a third (36.8%) of proxy respondents were friends or family co-resident with the service user. The majority of proxy respondents (57.3%) were people outside of the household or residential home other than a paid carer. (These are most likely unpaid carers who are not co-resident with the care recipient but may include other groups: for example, advocates) [63]. This raises the question of whether the adaptation of a quality of life tool for proxy-report should seek to target the majority group of proxy respondents (that is, unpaid carers) or whether it should seek to improve engagement with paid carers as potential proxy respondents. At the very least, we should seek to identify who the proxy respondent is and be aware of the potential bias linked to different motivations of respondents.

While the non-equivalence of self-report and proxy report is acknowledged, some studies have found that the systematic difference between average self-reported and proxy-reported quality of life score is modest [16, 18, 41, 53, 64–66] or not significant [55, 67–69]. In which case, if the development of a proxy version of a quality of life instrument was primarily motivated by the minimisation of ‘proxy bias’ or the reduction of the inter-proxy gap [45], it would be reasonable to question the usefulness of developing a proxy version if the inter-proxy gap is negligible in large survey data collections. In this study, however, we have identified that the development of an instrument adapted for completion by proxy respondents could address the reluctance to answer questions on behalf of somebody else, and also improve the face validity of the instrument and the acceptability of its use. This may also enable wider participation in data collection that may influence policy, planning and administration of social care services and enable individuals, who would otherwise be excluded, to have a voice.

The study has some limitations. Whilst the findings may be applicable to the use of proxy tools more generally, participants were asked specifically about their views of answering the ASCOT on behalf of a person they care for. A further limitation of this study is, therefore, that it did not consider proxy respondents other than paid and unpaid carers (for example, advocates or other third sector staff or volunteers).

## Conclusion

This study has shown some of the challenges involved in assessing outcomes by proxy and also explored some potential ways these can be mitigated. The approach was to explore the views of paid carers and unpaid carers. Participants acknowledged some of the difficulties they faced as a proxy respondent, such as whose perspective they were being asked to provide (their own or that of the care recipient) and outlined the potential impact of differing proxy perspectives and motivations, which could lead to bias. Participants described having greater difficulty answering questions that were perceived to be ‘more abstract’, such as dignity, than those perceived to have more tangible cues, such as whether someone had had food and drink.

We argue that specific proxy questionnaires should be developed that use various framing questions to help people minimise bias. A number of provisions for proxy respondents were proposed to improve face validity and acceptability of completing a proxy questionnaire (from the viewpoint of ‘giving people a voice’); and to ensure that any potential bias is reduced in the design of the questionnaire. These included: providing two sets of response options for each proxy perspective (one for the proxy and from the view of the care recipient); a comments box to help people explain why they have selected a given response option (especially where these indicate unmet need); and providing clear guidance setting out exactly what is expected of the proxy respondent and how they should complete the questionnaire.

Moreover, both paid and unpaid carers agreed that any person acting as a proxy would need to be very familiar with the care recipient, as well as their needs and care provision. It would be important to record the relationship between the proxy and the care recipient in the proxy questionnaire.

The findings highlight the benefits of developing and testing proxy measures in a robust way (as opposed to making simple grammatical changes to a standard version) to widen participation in social care research.

## Additional files

Additional file 1: Proxy Interview Schedule for carers Topic guide for interviews with carers, Interview guide used for data collection. (DOCX 19 kb)

## Abbreviations

ASCOT: Adult Social Care Outcomes Toolkit
PROMs: Patient reported outcome measures
SCRQoL: Social care-related quality of life
SCT4: Self-completion, four-level questionnaire
