# Supplementary material for: Exploring the views of being a proxy from the perspective of unpaid carers and paid carers: developing a proxy version of the Adult Social Care Outcomes Toolkit (ASCOT)
Source: BMC Health Serv Res. 2019 Mar 29;19:201. doi: 10.1186/s12913-019-4025-1 (PMC6440097; doi:10.1186/s12913-019-4025-1)
Supplement: Supplementary file 1 — Proxy Interview Schedule for carers Topic guide for interviews with carers, Interview guide used for data collection. (DOCX 19 kb) [file 12913_2019_4025_MOESM1_ESM.docx]

## Topic guide for interviews with carers

## QORU Engagement – proxy version of ASCOT

**Notes for interviewer**

**Aim of project:** To develop and test a Carer version of the ASCOT that is suitable for someone that knows someone that uses social care services but would not normally be able to complete a questionnaire themselves.

**Aims of interview:** Would the carer feel able to answer the ASCOT domain questions on behalf of the person they care for or support? Why/why not?

**Introductions**

- **Introduce self and reiterate purpose of research (check they have read participant information sheet).**

I work at the University of Kent in a department called the Personal Social Services Research Unit or PSSRU for short and most of the work we do is about social care - the kind of support provided to [care recipient] and the support that people get in residential homes, nursing homes and care homes and so on - and how much it improves people’s lives.

- **Talk about ASCOT and current project**

One of the ways we look at how social care improves people’s lives is by looking at the answers people give to a survey called the Adult Social Care Survey. The Adult Social Care Survey is a questionnaire that is sent out, by post, once a year by local authorities across England to people who get social care services, either at home or in care homes. Each local authority sends out around 1000 questionnaires to a random group of people who get social care services in that area. The questionnaire asks lots of questions about all aspects of the care people receive and how it helps them (or not). People who want to and are able to complete the questionnaire send it back to their local authority and then it is all put together for the whole of England and the Government then analyses the results to see how well services are doing.

One part of the Adult Social Care Survey was designed by people in our department at the University of Kent. It is a set of questions about how care services help people in 8 particular areas of their lives and is known as the Adult Social Care Survey Outcomes Toolkit, or ASCOT for short. These questions have been designed so the answers people give to them can be added up to give them a score which represents what we call their ‘social care related quality of life’. Local authorities use this information as a way of understanding more about how services are working in their area and the Department of Health uses this information to look at how services are working across the whole of England. Some of the ASCOT questions are part of the government’s Adults Social Care Outcomes Framework (or ASCOF), which you may have heard of. This is what the Government uses to measure how well social care services are doing.

Although lots of people currently complete the Adult Social Care survey (over 150,000 in 2012) and the ASCOT questions that are part of it, lots more people who get social care services are not well enough or able to complete a questionnaire and give their views in that way. But we really want to know their views or at least get information from someone who knows them well and/or cares for them so we know how well services are working for them. And so the reason I’m here today is that we are trying to develop some new ASCOT questions that professional carers, family carers or friends could answer for someone who can’t do this themselves. So I would like to talk to you about the areas ASCOT asks questions about, show you how we ask service users themselves about these areas at the moment and then ask how easy they would be for you to answer on behalf of the person that you support and what we could change to make the questions better for you and easy to answer.

- **Introduce digital recorder**
- **Stress confidentiality**
- **Seek signed consent**

[start recording]

## Interview

### Background

- **Tell me a bit about the circumstances that have led to you being a carer.**

PROBE:

- - - How long have you been a carer?
    - Cared-for person?
    - Their health condition?
- **Tell me a bit about the kind of care you give and the things you do for the cared-for person.**

PROBE:

- - - Medication
    - Food and drink
    - Personal care

### Instructions

As I said, there are 8 questions we are looking at that relate to 8 areas of people’s lives. These areas have been chosen by lots of people - social care service users - that we have spoken to over the past 4 years or more - we didn’t just come up with them! So now I’d like to show you some of the questions and ask you what you think of them. We might not have time to do all 8 questions but that’s fine - we will see how far we get. This is really just to get your ideas and views on the questions, to help us think of the best way to ask them, and so there are no right or wrong answers. Is that okay? Are there any questions you’d like to ask? Feel free to ask any you have as we go along too.

So imagine that the person you support and care for, receives a questionnaire through the post. The questionnaire contains lots of questions about how that person feels about their life and the care and services they receive, that includes care from you and from other services. The person can’t answer for themselves and they have asked you to do this. The questionnaire says it is okay for someone else to answer on the cared-for person’s behalf, you just have to say so at the end of the questionnaire.

### Food & Drink

- **This is how we ask people a question about food and drink (Read out question and use showcard)**

Thinking about the person you support, would you be able to answer a question like that on their behalf?

**[If yes:]**

- - What answer would you give?
  - Why would you give that answer?
  - What would you/are you thinking about when you answer it?
  - Would you be able to involve the cared-for person in answering it?

**[If no:]**

- Why would it be difficult to answer?

PROBE:

- - - Would you feel comfortable answering on someone else’s behalf?
    - Do you think you would you have a different opinion to them?
      - What would this be?
      - Why?
    - Would you prefer to answer it from your viewpoint or from the viewpoint of the person you support? Or Both? Would it be difficult to answer from their viewpoint?
    - How might you change the wording of this question to make it easier for someone else to answer or easier for you to answer?
    - Any other comments or questions?

### Safety

- **This is how we ask people a question about safety (Read out question and use showcard)**

Thinking about the person you support, would you be able to answer a question like that on their behalf?

**[If yes:]**

- - What answer would you give?
  - Why would you give that answer? What would you/are you thinking about when you answer it?
  - Would you be able to involve the cared-for person in answering it?

**[If no:]**

- Why would it be difficult to answer?

PROBE:

- - - Would you feel comfortable answering on someone else’s behalf?
    - Do you think you would you have a different opinion to them?
      - What would this be?
      - Why?
    - Would you prefer to answer it from your viewpoint or from the viewpoint of the person you support? Or Both? Would it be difficult to answer from their viewpoint?
    - How might you change the wording of this question to make it easier for someone else to answer or easier for you to answer?
    - Any other comments or questions?

### Social Participation

- **This is how we ask people a question about social participation (Read out question and use showcard)**

Thinking about the person you support, would you be able to answer a question like that on their behalf?

**[If yes:]**

- - What answer would you give?
  - Why would you give that answer?
  - What would you/are you thinking about when you answer it?
  - Would you be able to involve the cared-for person in answering it?

**[If no:]**

- Why would it be difficult to answer?

PROBE:

- - - Would you feel comfortable answering on someone else’s behalf?
    - Do you think you would you have a different opinion to them?
      - What would this be?
      - Why?
    - Would you prefer to answer it from your viewpoint or from the viewpoint of the person you support? Or Both? Would it be difficult to answer from their viewpoint?
    - How might you change the wording of this question to make it easier for someone else to answer or easier for you to answer?
    - Any other comments or questions?

### Dignity

- **This is how we ask people a question about dignity (Read out question and use showcard)**

Thinking about the person you support, would you be able to answer a question like that on their behalf?

**[If yes:]**

- - What answer would you give?
  - Why would you give that answer?
  - What would you/are you thinking about when you answer it?
  - Would you be able to involve the cared-for person in answering it?

**[If no:]**

- Why would it be difficult to answer?

PROBE:

- - - Would you feel comfortable answering on someone else’s behalf?
    - Do you think you would you have a different opinion to them?
      - What would this be?
      - Why?
    - Would you prefer to answer it from your viewpoint or from the viewpoint of the person you support? Or Both? Would it be difficult to answer from their viewpoint?
    - How might you change the wording of this question to make it easier for someone else to answer or easier for you to answer?
    - Any other comments or questions?

### Occupation

- **This is how we ask people a question about occupation (Read out question and use showcard)**

Thinking about the person you support, would you be able to answer a question like that on their behalf?

**[If yes:]**

- - What answer would you give?
  - Why would you give that answer?
  - What would you/are you thinking about when you answer it?
  - Would you be able to involve the cared-for person in answering it?

**[If no:]**

- Why would it be difficult to answer?

PROBE:

- - - Would you feel comfortable answering on someone else’s behalf?
    - Do you think you would you have a different opinion to them?
      - What would this be?
      - Why?
    - Would you prefer to answer it from your viewpoint or from the viewpoint of the person you support? Or Both? Would it be difficult to answer from their viewpoint?
    - How might you change the wording of this question to make it easier for someone else to answer or easier for you to answer?
    - Any other comments or questions?

### Personal cleanliness and comfort

- **This is how we ask people a question about personal cleanliness and comfort (Read out question and use showcard)**

Thinking about the person you support, would you be able to answer a question like that on their behalf?

**[If yes:]**

- - What answer would you give?
  - Why would you give that answer?
  - What would you/are you thinking about when you answer it?
  - Would you be able to involve the cared-for person in answering it?

**[If no:]**

- Why would it be difficult to answer?

PROBE:

- - - Would you feel comfortable answering on someone else’s behalf?
    - Do you think you would you have a different opinion to them?
      - What would this be?
      - Why?
    - Would you prefer to answer it from your viewpoint or from the viewpoint of the person you support? Or Both? Would it be difficult to answer from their viewpoint?
    - How might you change the wording of this question to make it easier for someone else to answer or easier for you to answer?
    - Any other comments or questions?

### Control

- **This is how we ask people a question about control (Read out question and use showcard)**

Thinking about the person you support, would you be able to answer a question like that on their behalf?

**[If yes:]**

- - What answer would you give?
  - Why would you give that answer?
  - What would you/are you thinking about when you answer it?
  - Would you be able to involve the cared-for person in answering it?

**[If no:]**

- Why would it be difficult to answer?

PROBE:

- - - Would you feel comfortable answering on someone else’s behalf?
    - Do you think you would you have a different opinion to them?
      - What would this be?
      - Why?
    - Would you prefer to answer it from your viewpoint or from the viewpoint of the person you support? Or Both? Would it be difficult to answer from their viewpoint?
    - How might you change the wording of this question to make it easier for someone else to answer or easier for you to answer?
    - Any other comments or questions?

### Accommodation cleanliness and comfort

- **This is how we ask people a question about accommodation cleanliness and comfort (Read out question and use showcard)**

Thinking about the person you support, would you be able to answer a question like that on their behalf?

**[If yes:]**

- - What answer would you give?
  - Why would you give that answer?
  - What would you/are you thinking about when you answer it?
  - Would you be able to involve the cared-for person in answering it?

**[If no:]**

- Why would it be difficult to answer?

PROBE:

- - - Would you feel comfortable answering on someone else’s behalf?
    - Do you think you would you have a different opinion to them?
      - What would this be?
      - Why?
    - Would you prefer to answer it from your viewpoint or from the viewpoint of the person you support? Or Both? Would it be difficult to answer from their viewpoint?
    - How might you change the wording of this question to make it easier for someone else to answer or easier for you to answer?
    - Any other comments or questions?

**Is there anything else you would like to add?**

## Check: would they like to see a transcript of the interview?
